# Altered Functional Connectivity of Cognitive-Related Cerebellar Subregions in Alzheimer’s Disease

**DOI:** 10.3389/fnagi.2017.00143

**Published:** 2017-05-16

**Authors:** Weimin Zheng, Xingyun Liu, Haiqing Song, Kuncheng Li, Zhiqun Wang

**Affiliations:** ^1^Department of Radiology, Dongfang Hospital, Beijing University of Chinese MedicineBeijing, China; ^2^Department of Neurology, Xuanwu Hospital of Capital Medical UniversityBeijing, China; ^3^Department of Radiology, Xuanwu Hospital of Capital Medical UniversityBeijing, China; ^4^Beijing Key Laboratory of Magnetic Resonance Imaging and Brain InformaticsBeijing, China

**Keywords:** Alzheimer’s disease, resting state fMRI, functional connectivity, cerebellum, subregions, neuroimaging

## Abstract

Alzheimer’s disease (AD) is the most common cause of dementia. Previous studies have found disrupted resting state functional connectivities (rsFCs) in various brain networks in the AD patients. However, few studies have focused on the rsFCs of the cerebellum and its sub-regions in the AD patients. In this study, we collected resting-state functional magnetic resonance imaging (rs-fMRI) data including 32 AD patients and 38 healthy controls (HCs). We selected two cognitive-related subregions of the cerebellum as seed region and mapped the whole-brain rsFCs for each subregion. We identified several distinct rsFC patterns of the two cognitive-related cerebellar subregions: default-mode network (DMN), frontoparietal network (FPN), visual network (VN) and sensorimotor network (SMN). Compared with the controls, the AD patients showed disrupted rsFCs in several different networks (DMN, VN and SMN), predicting the impairment of the functional integration in the cerebellum. Notably, these abnormal rsFCs of the two cerebellar subregions were closely associated with cognitive performance. Collectively, we demonstrated the distinct rsFCs patterns of cerebellar sub-regions with various functional networks, which were differentially impaired in the AD patients.

## Introduction

Alzheimer’s disease (AD) is the most common cause of dementia manifested as progressive memory deficits and cognitive impairment (Seeley et al., 2009; Pievani et al., 2011a), which may be caused by the deposition of amyloid-β plaques, neurofibrillary tangles and neuronal loss (Braak and Braak, 1991; Kandimalla et al., 2011, 2014). These AD pathological changes attack the cognitive related specific regions and disrupt the connectivity among these regions, which leads to the cognitive decline. Therefore, AD is regarded as a disconnection syndrome (Buckner et al., 2009). However, the mechanism is not very clear at present.

Resting-state functional magnetic resonance imaging (rs-fMRI) has been increasingly utilized to explore neural networks by investigating the brain low frequency fluctuations in the blood-oxygen level-dependent (BOLD) signals (Biswal et al., 1995; Zhang and Raichle, 2010). By using fMRI technique, previous AD studies have demonstrated that the disrupted resting-state functional connectivities (rsFCs) mainly concentrated on several key regions of neuronal degeneration in AD, such as the anterior hippocampus (BA 27/28; Wang et al., 2006), posterior cingulate cortex (BA 23/29/31; Greicius et al., 2004; Zhang et al., 2010), prefrontal cortex (BA 8/9/10; Wang et al., 2007; Dai et al., 2012), inferior parietal lobe (BA 39/40; Wang et al., 2015) and thalamus (Wang et al., 2012). Besides these regions, cerebellum is recently regarded as an important cognitive-related region in AD. The cerebellum has traditionally been considered as a reference region in AD since it has been reported to provide unbiased estimations for intensity normalization (Mevel et al., 2007; Chételat et al., 2008). However, the idea that the cerebellum remains unaffected by AD is challenged by several recent studies. For example, gray matter (GM) atrophy of the cerebellum was detected in AD in several neuroimaging studies (Thomann et al., 2008; Bas et al., 2009). In addition, the AD patients exhibited significantly lower cerebral blood flow (CBF; Hauser et al., 2013), abnormal activation during Stroop tests (Kaufmann et al., 2008) and impaired rsFCs of the cerebellum (Wang et al., 2007; Zhang et al., 2014) in several studies. Furthermore, the AD pathological changes have now been revealed in the cerebellum including deposits of amyloid-β plaques, neurofibrillary tangles, increased microglia and so on (Larner, 1997; Ciavardelli et al., 2010; Sepulveda-Falla et al., 2011). These studies raised the interest of cerebellum which may be a vulnerable region in the AD patients. Thus, it is extremely important to ascertain the intrinsic rsFCs pattern of the cerebellum in AD.

The cerebellum is a region characterized with complex structure and function. Anatomically, the cerebellum is linked to the cerebrum through three cerebellar peduncles. It has efferent and afferent fibers between the vermis, hypothalamus and limbic system (Strata, 2015). Functionally, based on the anatomical links, the cerebellum is connected to several brain networks, which is involved in multimodal functions such as cognitive, emotional and sensorimotor processing (Schmahmann et al., 2007; Stoodley and Schmahmann, 2009; O’Reilly et al., 2010). Interestingly, several recent fMRI studies found the consistent rsFCs between the cerebellar sub-regions (lobule IX and Crus II) and the cognitive-related network regions. However, the results are not very consistent. Habas et al. (2009) reported lobule IX was linked with default mode network (DMN). However, Krienen and Buckner (2009) emphasized on Crus II and regarded it as an important role in DMN. Other studies from Sang et al. (2012) and Li et al. (2013) found the Crus II was typically connected with frontoparietal network (FPN) and the lobule IX was mainly connected with the DMN. Therefore, we sought to extend prior work to determine actual connectivity patterns of the cerebellar cognitive related subregions including the lobule IX and Crus II.

Considering the heterogeneity of the cerebellum and its association with DMN, it is extremely important to ascertain the intrinsic rsFCs of the cerebellar cognitive-related subregions in AD. Until now, only a few previous resting state studies focused on cerebellar connectivity in the early stage of AD patients (Bai et al., 2011; Castellazzi et al., 2014). One study applied independent component analysis (ICA) to analyze the cerebellum-related networks in the AD patients (Castellazzi et al., 2014). Although ICA can decompose BOLD data into independent components using statistical method, there are still some challenges for the ICA method. For example, there were no priori criteria to identify the number of independent components in BOLD data, which contributed to the final results in a high degree (Fox and Raichle, 2007). Another rs-fMRI study explored regional cerebellar activation and rsFC in early risk state of AD (Bai et al., 2011). However, the study only selected part of lobule IX as seed region to detect the cerebellar connectivity. Furthermore, the BOLD data analysis was based on the 1.5T MRI which might mask some subtle changes in the results. To the present, no studies have reported altered patterns of intrinsic cerebellum subregional connectivity (Crus II and lobule IX) in AD using 3T MRI.

Here, we aim to investigate the intrinsic rsFCs of the cerebellar cognitive related subregions in the AD patients by using rs-fMRI, and to explore the association between the abnormal cerebellar rsFCs changes and patients’ cognitive performances. In order to explain that the results were not caused by the regional GM atrophy, we performed voxel-based morphometry (VBM) analysis and used the GM as nuisance covariate in the study. We hypothesized that the AD patients would show differentially disrupted rsFCs of cerebellar sub-regions, which were independent of GM atrophy and closely associated with cognitive performances.

## Materials and Methods

### Subjects

Seventy-five subjects including 34 AD patients and 41 age and gender matched healthy controls (HCs) were recruited and participated in the study at the memory clinic of Xuanwu Hospital. All the subjects were right-handed. The study was approved by the Medical Research Ethics Committee of Xuanwu Hospital. All subjects provided written informed consent prior to participation, consistent with the Declaration of Helsinki. The details of the consent form included the study’s aim, inclusion and exclusion criteria, procedures, potential harm and benefits, medical care, privacy rights, and withdrawal process. They were informed of their right to discontinue participation at any time. During the preprocessing, we excluded five subjects (two AD patients and three HCs). Table 1 showed the clinical information for the remaining 70 participants, including 32 AD patients and 38 HCs. All the AD patients underwent a complete physical and neurological examination standard laboratory tests, and an extensive battery of neuropsychological assessments, which included mini-mental state examination (MMSE), Clinical Dementia Rating (CDR), World Health Organization–University of California–Los Angeles Auditory Verbal Learning Test (WHO-UCLA-AVLT), the Extended Scale for Dementia (ESD), Montreal Cognitive Assessment (MoCA), Clock Drawing Task (CDT), Activity of Daily Living Scale (ADL), Functional Activities Questionary (FAQ), Hamilton Depression Scale (HAMD) and Hachinski Ischemic Score (HIS). The diagnosis of AD fulfilled the new research criteria for possible or probable AD (Dubois et al., 2007, 2010).

**Table 1:** **Demographic and neuropsychological test**.

	AD (*n* = 32)	HC (*n* = 38)	*P* Value
Age (years)	52 –86 (71.25 ± 8.63)	50 –86 (68.39 ± 7.78)	<0.15^a^
Gender (male/female)	14/18	13/25	<0.41^b^
CDR	0.5 (*n* = 14), 1 (*n* = 18)	0	–
MMSE	10 –25 (18.56 ± 3.99)	28 –30 (28.63 ± 0.67)	<0.001^a^
AVLT	8 –24 (14.81 ± 4.12)	39 –52 (44.42 ± 2.74)	<0.001^a^
ESD	107 –200 (155.33 ± 26.48)	180 –248 (227.74 ± 15.68)	<0.001^a^
MoCA	8 –19 (14.94 ± 3.23)	27 –30 (28.63 ± 0.67)	<0.001^a^
CDT	3 –8 (6.13 ± 1.43)	8 –9 (8.71 ± 0.46)	<0.001^a^
ADL	22 –45 (30.41 ± 7.21)	20 –22 (21.08 ± 0.78)	<0.001^a^
FAQ	4 –11 (6.25 ± 1.70)	0 –2 (0.55 ± 0.76)	<0.001^a^
HAMD	0 –3 (1.06 ± 1.08)	0 –3 (0.61 ± 1.00)	<0.07^a^
HIS	0 –3 (1.16 ± 0.77)	0 –3 (1.13 ± 1.07)	<0.91^a^

*Data are presented as the range of minimum—maximum (mean ± SD). Abbreviations: AD, Alzheimer’s disease; ADL, Activity of Daily Living Scale; WHO-UCLA-AVLT, World Health Organization–University of California—Los Angeles Auditory Verbal Learning Test; CDR, Clinical Dementia Rating; CDT, Clock Drawing Task; ESD, The Extended Scale for Dementia; FAQ, Functional Activities Questionary; HAMD, Hamilton Depression Scale; HCs, healthy controls; HIS, Hachinski Ischemic Score; MMSE, Mini-Mental State Examination; MoCA, Montreal Cognitive Assessment. ^a^The P value was obtained by two-sample two-tailed t-test; ^b^The P value was obtained by two-tailed Pearson chi-square test*.

The criteria for HCs were as follows: (a) no neurological or psychiatric disorders such as stroke, depression, epilepsy; (b) no neurological deficiencies such as visual or hearing loss; (c) no significant abnormal findings such as infarction or tumor in conventional brain MR imaging; (d) no cognitive complaints; (e) MMSE score of 28 or higher; and (f) CDR score of 0.

### Data Acquisition

MRI data were collected using a SIEMENS Trio 3-Tesla scanner (Siemens, Erlangen, Germany). Sponge pads were placed to limit head motion. The subjects were asked to keep their eyes closed and think of nothing in particular. The rsfMRI scans were performed using an echo-planar imaging (EPI) sequence: repetition time (TR)/echo time (TE) = 2000 ms/40 ms, flip angle (FA) = 90°, field of view (FOV) = 24 × 24 cm, matrix = 64 × 64, thickness = 4 mm, gap = 1 mm, voxel size = 3.75 × 3.75 × 4 mm^3^, bandwidth = 2232 Hz/pixel. Each volume comprised of 28 axial slices and 239 time points were collected. The scan lasted for 478 s. The configuration data were collected by a 3D sagittal T1-weighted magnetization-prepared rapid gradient echo (MPRAGE) sequence: TR/TE = 1900 ms/2.2 ms, inversion time (TI) = 900 ms, FA = 9°, matrix = 256 × 256, slices = 176, thickness = 1.0 mm, no gap, voxel size = 1 × 1 × 1 mm^3^. All the subjects had not fallen asleep according to a simple questionnaire after the scan.

### Data Preprocessing

Data preprocessing were performed using the Statistical Parametric Mapping (SPM^1^) and Data Processing Assistant for rs-fMRI (DPARSF^2^; Chao-Gan and Yu-Feng, 2010) toolkits. Briefly, preprocessing included removal of the first 10 volumes, slice timing and head motion correction. To spatially normalize the fMRI data, the T1-weighted images were used to register the functional data to their corresponding anatomical image, and the resulting aligned T1 dataset was transformed into Montreal Neurological Institute (MNI) space (Ashburner and Friston, 2005). During the normalization, five subjects (two patients and three controls) were excluded. Then, the normalized functional images were created by transforming the T1 images to the customized T1 template. The inaccuracy of the spatial normalization of functional volumes caused by GM atrophy in the AD patients and HCs could be reduced by such a custom template-based registration procedure. The functional images were resampled to 3 mm isotropic voxels. Then these images were smoothed spatially (the full width-half maximum Gaussian kernel was set to 4 mm). In order to reduce the influence of low-frequency drifts and high-frequency physiological noise, linear detrending and temporal bandpass filtering (0.01–0.1 Hz) were applied. Finally, several nuisance variables were removed by multiple linear regression analysis, including six head motion parameters, cerebrospinal fluid and white matter. During image preprocessing, no excessive head motion was found in our subjects (defined by a translation greater than 3 mm or rotation greater than 3°).

### Definition of Cerebellar Subregions

**Figure 1:**
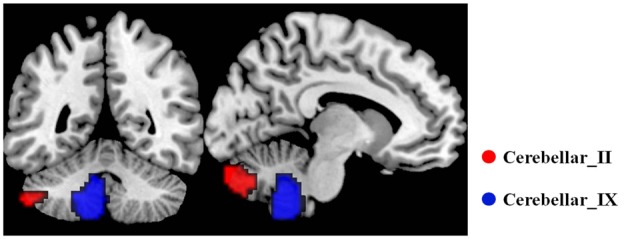
**The subregions of cerebellar**. In each hemisphere, two subregions were defined including the Crus II and posterior lobule IX using the probabilistic cerebellar atlas with a threshold of 50% minimum probability. Red color represents the Crus II, and green color represents the posterior lobule IX.

The bilateral cerebellar subregions of Crus II and posterior lobule IX were extracted from the Probabilistic cerebellar atlas (Diedrichsen et al., 2009) with a threshold of 50% minimum probability (Figure 1).

### Functional Connectivity Analysis of the Cerebellar Subregions

For each subject, the voxels of each cerebellar subregions were extracted and averaged to obtain the seed point reference time series. A correlation map was produced by computing the correlation coefficients between the reference time series and the time series from all the other brain voxels. Correlation coefficients were then converted to *z* values using Fisher’s *r*-to-*z* transform to improve the normality. For all the subjects, four *z-score* maps were obtained which represented the intrinsic rsFCs of the four cerebellar subregions (two for each hemisphere).

### VBM Analysis

Previous studies have reported significant atrophy of GM volume in the AD patients (Frisoni et al., 2002; Busatto et al., 2003). To eliminate the influences of GM atrophy on the rsFCs differences between the AD and HC group, we took voxel wise GM volume as covariate during the rsFC analysis (He et al., 2007; Wang et al., 2011). First, each subject’s GM volume map was estimated from the normalized T1 images by VBM method (VBM8 toolbox^3^; Ashburner and Friston, 2000). Then, we performed a two-sample *t* test between the AD and HC group, this procedure was to show GM atrophy in the AD patients.

### Statistical Analysis

To examine the within-group rsFCs of each cerebellar subregion for the AD patients and HCs, we performed one sample *t* tests on individual *z*-score maps for each cerebellar subregion. The statistical significance threshold was set to *P* < 0.05 with FWE corrected.

To assess the between-group differences of the whole-brain rsFCs of each cerebellar subregion, a two-sample *t* tests was performed with age, gender and GM volume being treated as covariates. The significance threshold was set to *P* < 0.001 with topo FDR corrected.

Then, partial correlation analysis was performed to explore the associations of the clinical variables with the rsFC strength for each cerebellar subregion in AD group, with age, gender as nuisance covariates (*P* < 0.05).

## Results

### Demographic and Neuropsychological Tests

Table 1 showed the clinical information of all the participants. We did not find the significant differences of age and gender between the AD and HC group (both *P* > 0.1). However, the AD group exhibited significantly lower MMSE, CDR, AVLT, ESD, MoCA, CDT, ADL and FAQ scores than the HC group (*P* < 0.0001).

### Gray Matter Atrophy in the AD Group

**Figure 2:**
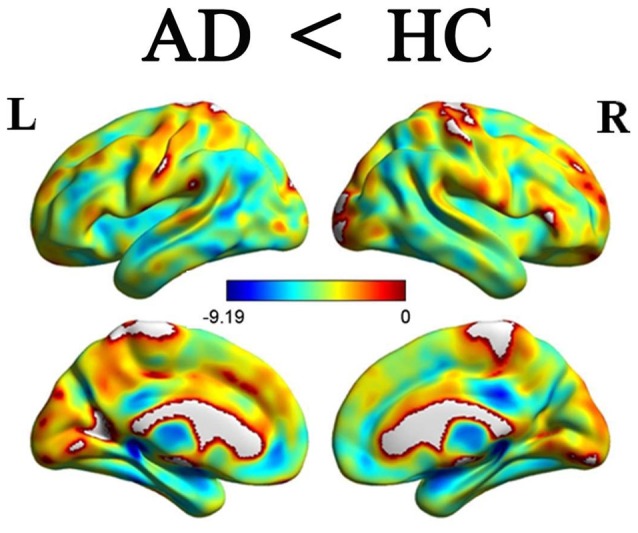
**Differences of GM volume between the AD patients and HCs *P* < 0.05, FDR corrected)**. Color bar represents *P* values. Compared with the HCs, the AD patients showed significant reduction of GM volumes. GM, gray matter; AD, Alzheimer’s disease; HCs, healthy controls.

When comparing with the HC group, the AD group showed significant and widespread GM atrophy, which was mainly involved in the lateral temporal cortex, medial temporal lobe, dorsal and medial frontal cortices, posterior cingulate cortex and lateral parietal regions, as well as the subcortical regions (Figure 2).

### Intragroup Functional Connectivity Maps of the Crus II and Posterior Lobule IX

**Figure 3:**
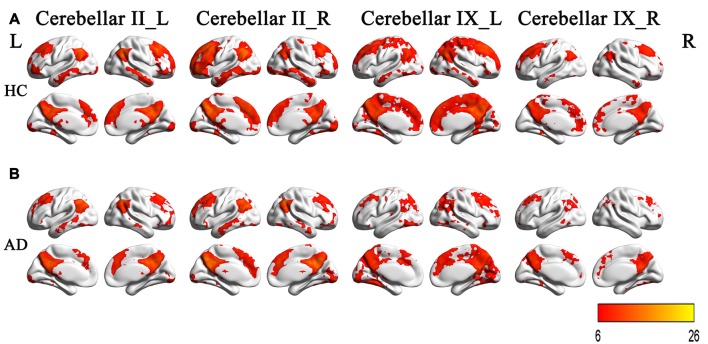
**The rsFCs patterns of the Crus II and posterior lobule IX in the HCs and AD patients. (A)** The rsFCs patterns of the Crus II and posterior lobule IX in HCs (*P* < 0.05, FWE corrected); **(B)** the rsFCs patterns of the Crus II and posterior lobule IX in the AD patients (*P* < 0.05, FWE corrected). Color bar represent for positive functional connections. Similar patterns for each subregion between the two groups were shown by visual inspection. The cerebellar crus II was mainly correlated with the DMN regions, FPN regions and VN regions. The posterior lobule IX showed positive connectivity with the several frontal and parietal regions, including the DMN, FPN, VN regions and part of SMN regions. rsFC, resting state functional connectivity; HCs, healthy controls; AD, Alzheimer’s disease; DMN, default-mode network; FPN, frontoparietal network; VN, visual network; SMN, sensorimotor network.

Figures 3A,B illustrated the rsFC maps for subregions of Crus II and posterior lobule IX within the HC and AD groups. By visual inspection, the two groups showed similar rsFC patterns for each cerebellar sub-region. The cerebellar crus II was mainly correlated with the DMN regions including the posterior cingulate cortex/precuneus(PCC/Pcu), medial prefrontal cortex (MPFC), lateral parietal cortices and temporal cortices. In addition, we also noticed the cerebellar crus II was associated with FPN regions including the dorsolateral prefrontal cortex (DLPFC) and posterior parietal cortex as well as visual network (VN) regions such as middle occipital region. The posterior lobule IX showed positive connectivity with the several frontal and parietal regions, including the DMN, FPN, VN regions and part of sensorimotor network (SMN) regions, including the bilateral precentral gyrus (PreCG) and postcentral gyrus (PostCG).

### Intergroup Functional Connectivity Maps of the Crus II and Posterior Lobule IX

**Figure 4:**
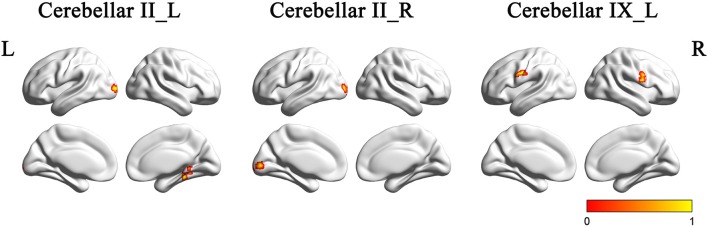
**The rsFCs differences between the AD patients and HCs (*P* < 0.001, topoFDR corrected)**. Color bar indicates the frequency that the region emerged between group comparisons. Compared with the HCs, the AD patients showed decreased rsFCs between the left Crus II and regions including the right PHG and left MOG, the right Crus II and the left MOG, the posterior lobule IX and SMN regions including PreCG and PostCG. rsFC, resting state functional connectivity; AD, Alzheimer’s disease; HCs, healthy controls; PHG, parahippocampus; MOG, middle occipital gyrus; SMN, sensorimotor network; PreCG, precentral gyrus; PostCG, postcentral gyrus.

Figure 4 and Table 2 showed the intergroup rsFC differences for each cerebellar subregion. Compared with the HC group, the AD group showed significantly decreased rsFCs between the left Crus II and regions including the right parahippocampus (PHG) and left middle occipital gyrus (MOG). In addition, the right Crus II showed significantly decreased rsFCs with the left MOG in the AD patients. When comparing the rsFC of the posterior lobule IX between the two groups, we found significantly decreased rsFCs in the AD patients in regions mainly located within the SMN regions including PreCG and PostCG. We didn’t find significantly increased rsFCs of the regions in the AD patients comparing to the HCs.

**Table 2:** **Regions showing AD-related resting state functional connectivity (rsFC) changes in cerebellar II and cerebellar IX**.

ROIs	Brain regions	Cluster voxels	MNI coordinates (mm)			Maximum *Z*
			*x*	*y*	*z*
L. CB_II	R.PHG	55	24	−45	−3	−4.83
	L.MOG	49	−21	−93	0	−4.68
R. CB_II	L.MOG	89	−21	−93	0	−4.87
L. CB_IX	R.PostCG	53	66	−3	21	−4.38
	L.PreCG	57	−57	6	27	−4.18

*Between groups differences were determined by two-sample t tests (P < 0.001, topoFDR corrected). Abbreviations: CB, cerebellar; L, left; MNI, Montreal Neurological Institute; MOG, middle occipital gyrus; PHG, parahippocampal gyrus; PostCG, postcentral gyrus; PreCG, precentral gyrus; R right; Z, statistical value of peak voxel showing differences between two groups; x, y, z, coordinates of primary peak locations in the MNI space*.

### Correlations between the rsFCs of Cerebellar Subregions and Clinical Performances in the AD Patients

**Figure 5:**
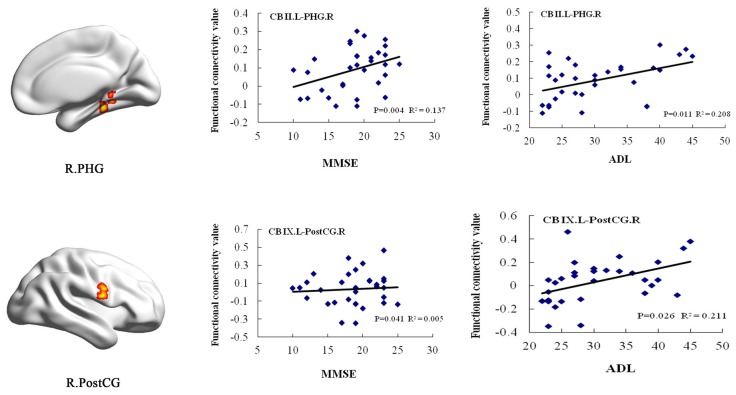
**Correlation of clinical variables and rsFCs of cerebellar subregions in the AD patients**. MMSE scores showed positive correlations with the cerebellar subregional rsFCs including the left Crus II and the right PHG, the left lobule IX and right PostCG. In addition, ADL scores showed positive correlations with the cerebellar subregional rsFCs including the left Crus II and the right PHG, the left lobule IX and right PostCG. ADL, activities of daily living; L, left; MMSE, mini-mental state examination; MOG, middle occipital gyrus; PHG, parahippocampal gyrus; PostCG, postcentral gyrus; R, right; rsFC, resting-state functional connectivity.

In the AD group, positive correlations were found between the MMSE scores and the cerebellum subregional rsFC, including connectivity between the left Crus II and the right PHG, the left lobule IX and right PostCG. In addition, we found positive correlations between the ADL scores and rsFCs between the left Crus II and right PHG, the left lobule IX and right PostCG (Figure 5).

## Discussion

### Major Findings

In the current study, we aim to investigate the intrinsic rsFCs of the cerebellar subregions in the AD patients by using rs-fMRI. By applying rsFC analysis to rs-fMRI data acquired from the HCs and AD patients, we observed two distinctive patterns of rsFCs for the cerebellar cognitive-related subregions: (1) the bilateral cerebellar Crus II exhibited rsFCs with the DMN, FPN and VN regions; and (2) the left lobule IX of the cerebellum was connected with the DMN, FPN, SMN and VN regions. Importantly, the AD patients showed differentially disrupted patterns of rsFCs in the specific subregions within different functional networks (DMN, VN and SMN), which were significantly associated with cognitive decline.

### Intragroup Functional Connectivity Maps of the Crus II and Posterior Lobule IX

The Crus II of the cerebellum was functionally connected to several DMN regions (PCC/Pcu, MPFC, IPL, lateral parietal cortices and temporal cortices) and FPN regions (DLPFC and posterior parietal cortex). In the previous studies, Krienen and Buckner (2009) demonstrated Crus II played an important role in DMN. Some other studies revealed that the Crus II was typically connected with the FPN regions (Sang et al., 2012; Li et al., 2013). In the current study, the crus II was mainly connected with both the DMN and FPN regions, which was consistent with the previous studies.

The lobule IX of the cerebellum was involved in the DMN, FPN, VN and SMN regions. Several previous studies (Habas et al., 2009; Sang et al., 2012; Li et al., 2013) reported the lobule IX was linked with the DMN regions, which was similar with the current study. In addition to the DMN regions, the present study also found that the lobule IX was connected with the FPN, SMN and VN regions.

### Intergroup Functional Connectivity Maps of the Crus II and Posterior Lobule IX

We found that the subregions of the cerebellum showed differentially disrupted rsFCs in the AD patients. Specifically, we observed disrupted rsFCs between the Crus II and the right PHG, bilateral MOG. In addition, we also found the decreased rsFCs between the posterior lobule IX and the SMN regions (PreCG and PostCG).

PHG is the critical node of the DMN. Several studies have reported the cortical thinning (Dickerson and Sperling, 2009), disrupted functional activity and connectivity (Greicius et al., 2004; Wang et al., 2011) of the DMN regions in the AD patients. Based on the previous findings, our findings provided further evidence for the destruction of the DMN in the AD patients, which was especially relevant to the disconnection between the Crus II of cerebellum and PHG. More importantly, we found that the rsFC values between the left Crus II and the right PHG were positively correlated with MMSE and ADL scores, suggesting the clinical relevance of functional disconnection in the AD patients.

Besides the PHG region, we also observed disrupted connectivity between the bilateral Crus II and the left MOG. The MOG is located in the primary visual cortex and play a critical role in visual cognition. By using fMRI method, Sala-Llonch et al. (2015) reported MOG region presented higher activity during the task of visuo-perceptual working memory. Using diffusion tensor imaging (DTI) and tractography, a previous study demonstrated that the structural disconnection in ventral occipital-temporal cortex contributed to the deficit in facial recognition (Thomas et al., 2009). Visual cognition deficits were consistently reported to accompany the development of AD (Cronin-Golomb, 1995; Bokde et al., 2006).

For the disrupted rsFC of SMN in the AD patients, some task-related fMRI studies have demonstrated reduced activation in the premotor cortex in the AD patients when performing the motor-related tasks (Agosta et al., 2010; Vidoni et al., 2012). Brier et al. (2012) found the significant functional abnormality of SMN in the AD patients by analyzing rs-fMRI data of 510 human subjects. In conjunction with these findings, we speculated that the AD patients might presented subtle motor impairment, which caused by the dysfunction of the SMN.

Pathologically, understanding the contribution of tau and amyloid-β pathology to cerebellum in AD would further expand our knowledge of the disease mechanisms (Liu et al., 2012). In some studies, the cerebellum was typically devoid of amyloid plaques and tau (Serrano-Pozo et al., 2011; Ni et al., 2013). While other studies revealed deposits of amyloid-β plaques, neurofibrillary tangles in the cerebellum in the AD patients (Larner, 1997; Ciavardelli et al., 2010; Sepulveda-Falla et al., 2011). Previous studies confirmed the majority of the cerebellum is connected to cerebral association networks (Buckner, 2013). Furthermore, our study revealed the disconnection between cerebellar sub-regions and several cerebral networks in the AD patients. We speculated that the disrupted rsFCs of the cerebellum in AD might be due to the pathological changes in the cerebellum. Further studies need to be performed in the future.

### Analysis of Age, Gender and GM Volume

In the current study, several factors including age, gender and GM volume would affect the final results. In collecting subjects, we paid high attention to these factors to match the two groups as well as possible. In the following rsFCs analysis, we used age, gender and GM volume as covariates to avoid the impact of the factors. As the results, we found the rsFCs changes of cerebellum in AD were independent of the above factors.

### Future Consideration

Several issues need to be considered in the future. First, in the current study, we focused on the rsFCs of the cerebellar sub-regions only by using the rsfMRI. Further studies that simultaneously combine the fMRI and DTI methods on the cerebellum would provide more information for the mechanism of AD. Second, to explore the early stage of AD patients would be helpful to find the valuable biomarker for diagnosis of the disease. For example, amnestic mild cognitive impairments (aMCI) and ApoE-4 allele carriers were paid more attention in the recent studies (Pievani et al., 2011b; Bai et al., 2012). Finally, to clarify the progressive changes of the connectivity in cerebellum subregions, a longitudinal research would be essential, which may provide a deep understanding of the AD pathophysiological mechanisms.

## Conclusion

We have identified the patterns of abnormal rsFCs in the two cognitive–related cerebellar subregions in the AD patients, which was involved in different functional brain networks such as DMN, VN and SMN. These findings added the new evidence for the disconnection syndrome of the disease, which might be helpful to search for the potential biomarker for early AD diagnosis in the future.

## Author Contributions

WZ and XL: the conception or design of the work; the acquisition, analysis and the interpretation of data for the work; drafting the work; final approval of the version to be published; agreement to be accountable for all aspects of the work; HS: the acquisition of data for the work; clinical evaluation; KL and ZW: the design of the work; revising the work; final approval of the version to be published; agreement to be accountable for all aspects of the work.

## Funding

This work was supported by the National Natural Scientific Foundation of China (Grant Nos. 81370037, 81571648 and 81471649).

## Conflict of Interest Statement

The authors declare that the research was conducted in the absence of any commercial or financial relationships that could be construed as a potential conflict of interest.

## Footnotes

^1^www.fil.ion.ucl.ac.uk/spm

^2^www.restfmri.net/forum/DPARSF

^3^http://dbm.neuro.uni-jena.de/vbm
